# Expression and Functional Relevance of Cannabinoid Receptor 1 in Hodgkin Lymphoma

**DOI:** 10.1371/journal.pone.0081675

**Published:** 2013-12-09

**Authors:** Alexander H. Benz, Christoph Renné, Erik Maronde, Marco Koch, Urszula Grabiec, Sonja Kallendrusch, Benjamin Rengstl, Sebastian Newrzela, Sylvia Hartmann, Martin-Leo Hansmann, Faramarz Dehghani

**Affiliations:** 1 Institute of Pathology, Justus-Liebig-University, Giessen, Germany; 2 Institute of Pathology, Goethe-University, Frankfurt, Germany; 3 Institute of Anatomy, Goethe-University, Frankfurt, Germany; 4 Institute of Anatomy, Leipzig University, Leipzig, Germany; 5 Department of Anatomy and Cell Biology, Martin-Luther-University Halle-Wittenberg, Halle, Germany; National Institutes of Health, United States of America

## Abstract

**Background:**

Cannabinoid receptor 1 (CB_1_) is expressed in certain types of malignancies. An analysis of CB_1_ expression and function in Hodgkin lymphoma (HL), one of the most frequent lymphomas, was not performed to date.

**Design and Methods:**

We examined the distribution of CB_1_ protein in primary cases of HL. Using lymphoma derived cell lines, the role of CB_1_ signaling on cell survival was investigated.

**Results:**

A predominant expression of CB_1_ was found in Hodgkin-Reed-Sternberg cells in a vast majority of classical HL cases. The HL cell lines L428, L540 and KM-H2 showed strong CB_1_-abundance and displayed a dose-dependent decline of viability under CB_1_ inhibition with AM251. Further, application of AM251 led to decrease of constitutively active NFκB/p65, a crucial survival factor of HRS-cells, and was followed by elevation of apoptotic markers in HL cells.

**Conclusions:**

The present study identifies CB_1_ as a feature of HL, which might serve as a potential selective target in the treatment of Hodgkin lymphoma.

## Introduction

The Endocannabinoid system consists of cannabinoid receptors, their endogenous, exogenous or synthetic ligands and the enzymes responsible for synthesis and degradation of endogenous ligands. So far, two types of cannabinoid receptors, namely CB_1_ and CB_2_ have been identified. Both belong to the superfamily of G-protein-coupled receptors [1], [2]. Activation of Cannabinoid receptors inhibit adenylate cyclase and cAMP production via Gi/o coupling but also activate phospholipase C, MAPK and phosphoinositide 3-kinase (PI3K) signaling pathways (Gq coupling) [3]. Cannabinoid receptor 1 (CB_1_) represents one of the most abundant G-protein-coupled receptors (GPCR) in the brain [4]. It binds exogenous and endogenous cannabinoids and is thereby associated with several physiological and pathological processes within the central nervous system [5]-[8] but is also linked to a variety of peripheral disorders, such as obesity, liver fibrosis [9]-[11] and cancer [2], [3], [12].

Recent evidence points to the involvement of CB_1_ in growth of human breast cancer cells [13]-[16]. High CB_1_-protein expression in prostate cancer cases was associated with an increased dedifferentiation of tumor cells and poor prognosis [17]. In contrast, patients suffering from hepatocellular carcinoma with high mRNA-expression of CB_1_ reportedly have a better prognosis [18]. SR141716 (Rimonabant®), an antagonist/inverse agonist of CB_1_, was shown to induce apoptosis in an *in vivo* model of colon carcinoma [19], [20]. The expression level of cannabinoid receptors in astrocytoma-cells has been reported as crucial for downstream signaling processing with consequences on cell-viability [21]. Thus, the effects of either activation or blocking CB_1_ depends on the tissue investigated and the local expression level of the receptor.

Hodgkin lymphoma (HL) is one of the most frequent lymphomas in the Western world mainly affecting young adults. Although the majority of HL cases at any clinical stage have a good prognosis under adequate therapy, still about 20% of patients develop highly mortal relapse [22]. HL is classified into classical HL (cHL) representing the largest subtype (95% of HL) and nodular lymphocyte predominant HL (NLPHL) which accounts for around 5% of the cases. The largest subentities of cHL are the nodular sclerosis (NS) and mixed cellularity (MC) types with 70% and 20% of the cases, respectively [23]. Histologically, cHL consists of B-cell derived mononuclear Hodgkin and multinucleated Reed-Sternberg (HRS-) cells surrounded by a vast non-neoplastic infiltrate [24].

p65 (also known as RelA) is a member of the NF-κB transcription factor family which is a key mediator in the TNF-signaling pathway contributing to a variety of cellular processes such as survival, proliferation and immune response. In HRS-cells, several mutations were identified leading to a loss of endogenous inhibitors of p65 such as I-κB [25]–[28] and A20 [29], [30]. Via bypassing apoptosis with subsequent cell death, high expression and activity of p65 in HRS cells [31] is regarded as a key mechanism in the pathogenesis of cHL [32].

Thus far, the functional relevance of CB_1_ has not been elucidated in HL. We therefore investigated the expression of CB_1_ in primary cases of different HL entities. We further determined the impact of CB_1_ specific agonist ACEA and the inverse agonist / antagonist AM251 on signal transduction cascades such as NF-κB/p65- or PI3K/Akt-pathway and on cell fate in HL-derived cell lines.

## Materials and Methods

### Tissue samples

All tissues samples were studied in accordance with the Helsinki declaration. Specimens, which were originally submitted for diagnostic purposes, were retrieved from the files of the Department of Pathology of the University of Frankfurt.

### Immunohistochemical staining

For immunohistochemical staining, 3 µm thick sections of fixed (5% [w/v] buffered formalin) and paraffin-embedded tissue samples were generated and deparaffinized. Antigen retrieval was performed by incubation in a microwave oven for 10 min in 1 mM EDTA (pH 8.0). Sections were exposed to a 3% (v/v) H_2_O_2_-methanol solution for 10 min, washed in water and blocked with Tris-buffered saline (TBS, 3% [w/v] bovine serum albumin, BSA) for 20 min at room temperature. Antibodies against N-terminal (1.65 µg/mL, #101500, Cayman Chemical, Ann Arbor, USA) or C-terminal CB_1_ (5 µg/mL, #10006590, Cayman Chemical) were added in TBS containing 3% (w/v) BSA for 16 h at 4°C. After washing with TBS, sections were incubated with rabbit specific biotinylated secondary antibody (DAKO, Hamburg, Germany) followed by horseradish peroxidase conjugated streptavidin (DAKO) with TBS-wash steps in between. Staining was developed with diaminobenzidine (DAB, DAKO) for 3 min and subsequent counterstain of nuclei was performed using Meyer’s haematoxylin (Applichem, Darmstadt, Germany).

Specific signals for N- and C-terminal CB_1_ in human hippocampus and in a case of nodular sclerosing HL were absent when antibody was preabsorbed using the corresponding CB_1_ immunizing peptides in equimolar concentrations confirming antibody specificity (Figure S1). Picture acquisition was performed using a Zeiss microscope (Zeiss, Göttingen, Germany) equipped with an Axio-Cam digital camera (Zeiss) at 100, 200 and 400 fold magnification. The analysis of CB_1_ immunoreactivity was independently performed by three pathologists. A case was rated positive when more than 30% of its tumor cells displayed immunoreactivity for CB_1_ as generally accepted.

### Fluorescence staining and confocal laser scanning microscopy

For fluorescent immuno-labeling, antigen retrieval and blocking was performed (see above) and antibodies against CB_1_ (see above) and CD3 (1∶100, #NCL-CD3-PS1, DAKO), CD20 (1∶100, #U7021, DAKO), CD30 (1∶100, #M0751, Novocastra, Berlin, Germany), CD68 (1∶100, #M0876, DAKO) or CD138 (1∶100, #M7228, DAKO) were added in TBS containing 3% (w/v) BSA for 16 h at 4°C. After washing with TBS, secondary antibodies conjugated to Alexa-488 or Alexa-568 (Invitrogen, Darmstadt, Germany) were added (1:500 in TBS 3% [w/v] BSA) for 1 h. After washing with TBS, nuclear staining was performed in aqueous 2-(4-amidinophenyl)-1H -indole-6-carboxamidine (DAPI) solution (2 µg/mL). Specimens were analyzed with a LSM 510 meta confocal laser scanning microscope (Zeiss). For visualization of CB_1_-labeled cells, monochromatic light at 488 nm and an emission bandpass filter of 505-530 nm was used. For detection of Alexa-568-labeled antigens (CD3, CD20, CD138 and CD68) monochromatic light at 543 nm and an emission bandpass filter of 585-615 nm was used. DAPI staining was detected using light at 405 nm and an emission bandpass filter of 470−490 nm. Confocal images were obtained at 100- and 400-fold magnification.

### Cell culture experiments

Cell lines were obtained from the *Deutsche Sammlung von Mikroorganismen und Zellkulturen GmbH* (DSMZ, Braunschweig, Germany) maintained in RPMI 1640 containing fetal bovine serum (FBS, PAA, Pasching, Austria) and penicillin–streptomycin mix (Gibco) at 37°C and 5% CO_2_. Peripheral blood B-lymphocytes were isolated from healthy donors and isolated by Ficoll-Paque PREMIUM (GE Healthcare, München, Germany) density centrifugation. CD19^+^ cells were separated by magnetic cell separation using the MACS system (Miltenyi Biotec, Bergisch Gladbach, Germany) and were maintained in RPMI with 10% (v/v) FBS containing penicillin-streptomycin.

*N*-(2-Chloroethyl)-5*Z*,8*Z*,11*Z*,14*Z*-eicosatetraenamide (ACEA) and *N*-(Piperidin-1-yl)-5-(4-iodophenyl)-1-(2,4-dichlorophenyl)-4-methyl-1*H*-pyrazole-3-carboxamide (AM251) were obtained from Tocris Bioscience (Bristol, UK), L-α-lysophospatidylinositol (LPI) was purchased from Sigma (Deisenhofen, Germany). A 10 mM stock solution of each reagent was prepared in Di-methyl sulfoxide (DMSO, Sigma) and stored at −20°C. Cell lines L428, L540, KM-H2 and Karpas 422 were stimulated with ACEA, AM251 or LPI at final concentrations of 0.3, 1.0, 3.0 and 10 µM and analyzed after 24 h, 72 h or 120 h.

### RT-PCR analyses

PCR was performed using a rotor-cycler (Rotor-Gene™ RG 6000; Corbett Research, Pty Ltd, Sydney, Australia). The reaction volumes contained 20 µL as follows: 10 µL PCR-MasterMix (Promega, Inc., Madison, WI, USA), 0.5 µL of upper and lower strand primer for CB_1_, CB_2_ and GPR55 (25 pM, Sigma Aldrich, Taufkirchen, Germany), 0.25 µL of Eva Green dye (Eva Green, Biotrend Chemicals, UC, Destin, FL, USA) and 6.75 µL of RNase free water (CB_1_ upper: CTCAGTCATTTTGAGCTCAGCC; CB_1_ lower: GCCATGTCACCTTTGATGTCTTC; CB_2_ upper: GCTCCTCATCTGTTGGTTCC; CB_2_ lower: TGACCATGGAGTTGATGAGGC; GPR55 upper: GGTGCTCTCCCTCCCATT; GPR55 lower: GCTCACCAGTAGCGGGTAAC; ß-Actin upper: ACTCCTACGTGGGCGACGAGG; ß-Actin lower: CAGGTCCAGACGCAGGATGGC). The PCR reaction consisted of 5 steps: initial denaturation at 95°C, followed by 40 cycles of denaturation at 94°C (30 sec), annealing at 64°C, elongation at 72°C (30 sec) and fluorescence detection at 80°C (15 sec). PCR products were loaded on 2% (v/v) agarose gels diluted in 1xMOPS (Carl Roth GmbH, Karlsruhe, Germany) buffer containing GelRed™ Nucleic Acid Gel Stain (Biotium, Hayward, CA, USA) and visualized under UV light.

### SDS-PAGE and Western blot analyses

Cell suspensions were centrifuged at 300 g for 5 min and pellets were lyzed in sample buffer (Invitrogen) containing 100 mM lithium dodecylsulphate (Sigma). Cell extracts were sonicated, heated for 10 min at 70°C and chilled on ice. Prior to detection of CB_1_ signals, the amount of each loaded cell line was adjusted to similar ß-actin immunosignal. Extracts consisting of about 10,000 L428 cells were used for each experiment. Samples were loaded on Bis/Tris gradient gels (Invitrogen), separation was performed with a current of 80 mA for 90 min. Gels were blotted onto polyvinylidenfluoride membrane (Millipore, Billerica, USA), washed with TBS (pH 7.6) containing 0.1% (v/v) Tween-20 (TBS-T, Sigma), incubated for 60 min in Rotiblock (Roth, Karlsruhe, Germany) and transferred to Rotiblock solution containing primary antibody against CB_1_ (0.5 µg/mL, #101500, Cayman Chemical), phosphorylated (P-) Erk1/2 (1:5000, #4370, Cell Signaling, Bad Nauheim, Germany), P-Ser473-Akt (1:5000, #4060, Cell Signaling), P-p38 MAPK (1∶3000, #9211, Cell Signaling), p65 (1∶5000, #4764, Cell Signaling), ß-actin (1∶40.000, #A5316, Sigma), cleaved caspase-3 (1∶3000, #9661, Cell Signaling), or full length caspase-3 (1∶5000, #9665, Cell Signaling) overnight at 4°C. Membranes were washed three times with TBS-T, and incubated with the appropriate secondary HRP-coupled antibodies against rabbit or mouse (both DAKO) in Rotiblock for 60 min. After another wash step with TBS-T, signal detection was performed using the ECL plus system (GE Healthcare), High Performance ECL chemiluminescence films (GE Healthcare) and Readymatic developer and fixer (Carestream Health Inc., NY, USA). Specificity of N-terminal antibody against CB_1_ was confirmed using equimolar concentration of immunizing peptide (Figure S2).

Signal intensities of the digitized images were analyzed using a combination of densitometry and volumetry as implemented in the QuantiScan software (BioSoft, Cambridge, UK) as described in detail before [33]. Each area/density value for a specific protein band was normalized against the corresponding ß-Actin signal of each extract.

### Cell viability assay

Cells were grown in 96 well tissue culture plates (1,000 cells in 0.1 mL RPMI 1640, 10% [v/v] FBS) and stimulated as indicated. After an incubation time of 120 h, 10 µL of 3-(4,5-dimethylthiazol-2-yl)-2,5-diphenyltetrazolium-bromide (MTT, Roche, Mannheim, Germany) was added and, after incubation for 4 h, 0.1 mL solubilization buffer (Roche) was added. After 16 h at 37°C in humidified atmosphere, absorbance was measured with an EL311SX microplate reader (Biotek Instruments, Winooski, USA) at 550 nm with the reference wavelength set to 690 nm.

### Flow cytometric analyses

100,000 cells were cultured in RPMI 1640 containing 10% (v/v) FBS in a 6 well culture plate and treated as indicated. Flow cytometric analysis was performed using a FACSCanto II Flow Cytometer (BD Bioscience, San Jose, USA). For each measurement, 10,000 events within the live gate were counted. Fluorescence distribution was displayed as dot plot analysis and the percentage of fluorescent cells in each quadrant was determined using DIVA-software (BD Bioscience).

To characterize externalization of phosphatidyl-serine and plasma membrane permeability, the AnnexinV-PE/7-AAD Apoptosis Detection kit (BD Pharmingen, Franklin Lakes, USA) was used according to the manufacturer’s instructions.

Cell cycle analysis was performed using Click-iT EdU Alexa-647 Flow Cytometry Assay (Invitrogen) according to the manufacturer’s instructions. Briefly, DNA synthesis was measured by incorporation of the Alexa-647 labeled thymidine analog 5-ethynyl-2’-deoxyuridine (EDU) and the DNA content was examined using a cell cycle sensitive dye.

### Statistical analysis

Data were analyzed using the GraphPad Prism 5.0 software (GraphPad, San Diego, CA, USA). For multiple group comparison one-way ANOVA algorithm was used, followed by the Bonferroni *post hoc* test. For comparison of two groups Student’s *t* test was performed. The criterion of significance was p<0.05.

## Results

### Expression of CB_1_ in cases of classical Hodgkin lymphoma and non-neoplastic lymphatic tissues

**Figure 1:**
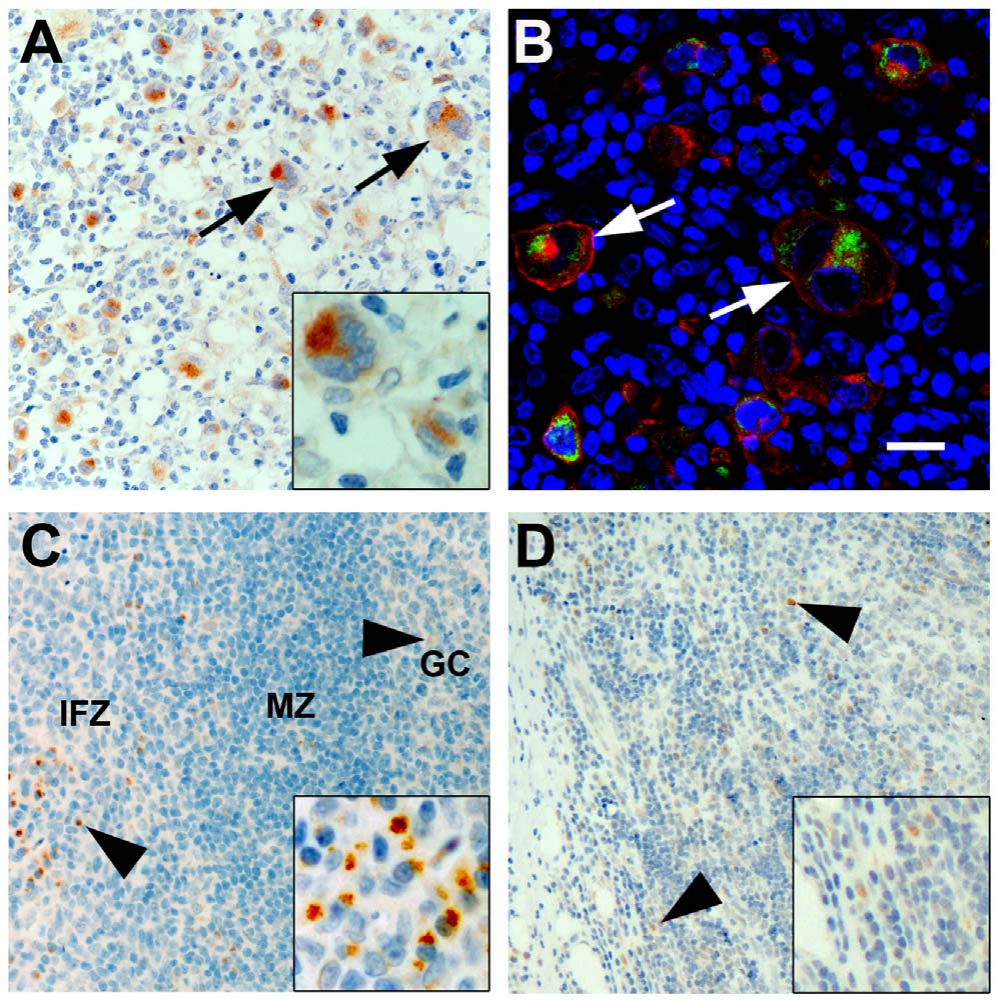
Expression of CB_1_-receptor in classical Hodgkin lymphoma and reactive non-neoplastic lymphatic tissues. A) Immunohistochemical staining of CB_1_–N in classical Hodgkin lymphoma showing strong expression of CB_1_ in HRS cells (arrows). B) Confocal image showing CB_1_ (green), CD30 (red) and DAPI-stained nuclei (blue) in cHL. Note the CB_1_ negativity in non-neoplastic infiltrate. C) In a case of reactive tonsillitis, CB_1_-positive cells (arrow heads) were found in the inter-follicular zone (IFZ) and to a lesser extent in germinal center (GC) and mantle zone (MZ). D) Few disseminated cells were found CB_1_-positive (arrow heads) in a case of lymphadenitis. Bars  =  20 µm.

**Figure 2:**
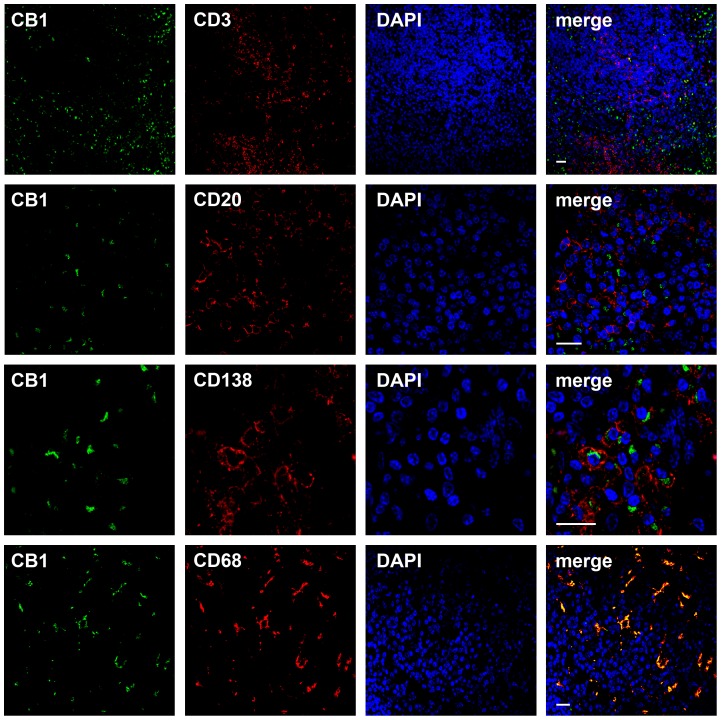
CB_1_ in reactive lymphoid tissue. Immunofluorescence staining and confocal imaging of a tonsil against CB_1_–N (green, left column), CD3, CD20, CD138 and CD68 (all in red, second left column). Nuclei were visualized using DAPI (blue, second right column). Right column represents merged images. CB_1_ signal was present in CD68^+^ macrophages and CD138^+^ plasma cells, not in CD3^+^ and CD20^+^ lymphocytes. Bars  =  20 µm.

To determine occurrence and localization of CB_1_ protein in Hodgkin lymphoma and normal lymphatic tissue, immunohistochemical staining with CB_1_-specific antibody was performed. Abundant CB_1_ protein was found in CD30^+^ HRS cells of cHL whereas the surrounding reactive, non-neoplastic lymphatic infiltrate was largely negative. The CB_1_-specific signal was located inside HRS cells, mainly with a perinuclear staining pattern (Figure 1A, B). In cases of tonsillitis and lymphadenitis, few cells displayed CB_1_-positivity in some of the germinal centers and inter-follicular zones (Figure 1C, D). To further characterize the positive cells in the reactive tissues, CB_1_-counterstaining was performed in a case of tonsillitis. CB_1_-specific signal was localized in the cytoplasm of CD138^+^ plasma cells and within branches of CD68^+^ macrophages while both CD3^+^ and CD20^+^ lymphocytes were found negative for CB_1_ (Figure 2).

### CB_1_-expression in B-cell lymphoma derived cell lines

**Figure 3:**
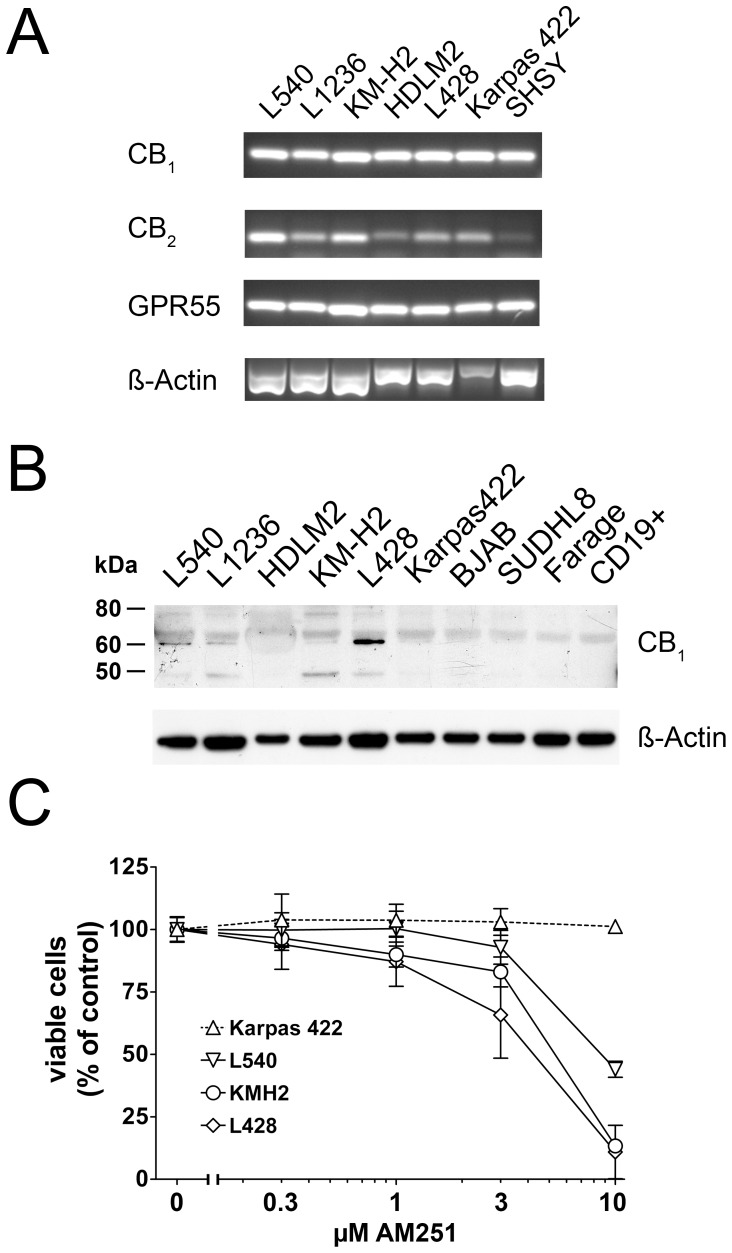
CB_1_-expression in B-cell derived cells and reduction of viability of cHL cells with AM251. A) Extracts of HL cell lines L540, L1236, KMH2, HDLM2, L428, as well as B-NHL cell line Karpas 422 and neuroblastoma derived cell line SHSY were used for mRNA analyses. After reverse transcription, cDNA templates were used to quantify mRNA transcripts of *Cnr1*, *Cnr2*, *GPR55* and *ß-actin*. B) Western blot analysis for CB_1_–N in cHL (L540, L1236, HDLM2, KM-H2 and L428), B-NHL-derived cell lines (Karpas 422, BJAB, SUDHL8, Farage) and isolated peripheral blood CD19^+^ B-lymphocytes. ß-actin signal served as loading control. C) Cell viability was determined in L428, L540, KM-H2 and Karpas 422 cells treated with the indicated concentrations of AM251 for 120 h using the MTT-assay. Reduced viability of cHL cell lines was observed whereas viability of Karpas 422 cells was not affected. Values represent means ± SD.

On the basis of our histopathological findings of CB_1_ expression in cases of lymphoma cases (Figure S3), we further investigated CB_1_ expression in HL cell lines L428, L540, L1236, HDLM2, KM-H2, as well as in the non-Hodgkin lymphomas (B-NHL) derived Karpas 422, BJAB, SUDHL8 and Farage cells using RT-PCR and Western blot analyses (Figure 3A,B). In PCR analyses, CB_1_ was detected in the neuroblastoma cell line SHSY as well as in all investigated lymphoma derived cells (Figure 3A). Interestingly, a moderate signal for the “peripheral” cannabinoid receptor CB_2_, was obtained in lymphoma cells, which was weaker in SHSY cells. All investigated cell extracts showed strong expression of GPR55. Since Hodgkin- and Reed-Sternberg cells of HL originate from B-cells, we included isolated CD19^+^ B-lymphocytes in the Western blot analyses. A band for CB_1_ at approximately 60 kDa was most prominent in L428 cells, lower in L540, L1236 and KM-H2. Two other bands were detected at around 50 and 80 kDa, both being most intense in KM-H2, followed by L1236, L428, L540 and to a lesser extent in Karpas422 cells. HDLM2, BJAB, SUDHL8, Farage cell lines and CD19^+^ cells were negative (Figure 3B). Different sizes obtained in Western blot analyses might be due to post-translational modification of the 50 kDa core protein, specificity of the used antibodies was verified by preabsorption. Next, functional relevance of CB_1_ was tested in L428, L540, KM-H2 as representative cell lines for HL and in Karpas 422 cell line representing B-NHL.

### AM251 impairs viability of HL cell lines

To test the functional relevance of detected CB_1_ on cell fate, cell lines were kept in culture medium containing 10% (v/v) FBS to provide optimal growth condition. The cell lines L428, L540, KM-H2 and Karpas 422 were treated with CB_1_ antagonist AM251 and viability was assessed using MTT-assay. Gallotta and colleagues observed a decrease in viability of Jurkat cells with an IC_50_ of around 12 µM using SR141716, a CB_1_-antagonist with an affinity to CB_1_ similar to AM251 [34]. Therefore, we tested viability of lymphoma cells using CB_1_ ligands at a maximum of 10 µM each.

Inhibition of CB_1_ by AM251 led to a significant decrease in viability of L428 cells at 3 µM (65.8±17.3%, p<0.05; n = 51) and 10 µM (10.9±10.7%, p<0.001; n = 39) after 120 h as compared to vehicle treated samples (Figure 3C). Furthermore, reduction of viability was observed in L540 cells (3 µM: 92.9±6.8%, p<0.05; 10 µM: 44.0±3.2%, p<0.001; n = 12) and KMH2 cells (1 µM: 90.0±5.0%, p<0.01; 3 µM: 83.0±6.0%; p<0.001; 10 µM: 13.4±2.1%, p<0.001, n = 12). Viability of Karpas 422 cells was not reduced at 3 µM (103±5.3%, p>0.05, n = 6) or 10 µM (101.2±1.9%, p>0.05, n = 6) when compared to controls.

Stimulation of L428 cells with ACEA did not significantly affect the viability at 3 µM (95.6±8.8%, p>0.05) but at 10 µM (82.9±9.8%, p<0.05; Figure S4A). Treating Karpas 422 with ACEA did not significantly reduce viable cell number at 3 µM (98.3±7.4%, p>0.05) or 10 µM (91.7±4.7%, p>0.05).

Next, we confirmed that AM251 induced effects on cell viability in L428 were due to inhibition of CB_1_ and not to activation of the GPCR GPR55, another target of AM251 [35] which was also detected in HL cell lines at mRNA level (Figure 3A). Therefore, GPR55-agonist LPI was applied as control. In comparison to vehicle treated cells, LPI had no significant effect on cell viability at 3 µM (101.5±8.7%, p>0.05, n = 12) but a small significant inhibitory effect at 10 µM (93.9±5.8%, p<0.05, n = 12) (Figure S4A).

To uncover the mechanisms behind the described decreased viability, further experiments were carried out in L428 cells, which displayed both, a strong CB_1_ immunosignal and a remarkable response in the viability assays.

### AM251 reduces p65, diminishes cells in S-phase and induces apoptosis in L428 cells

**Figure 4:**
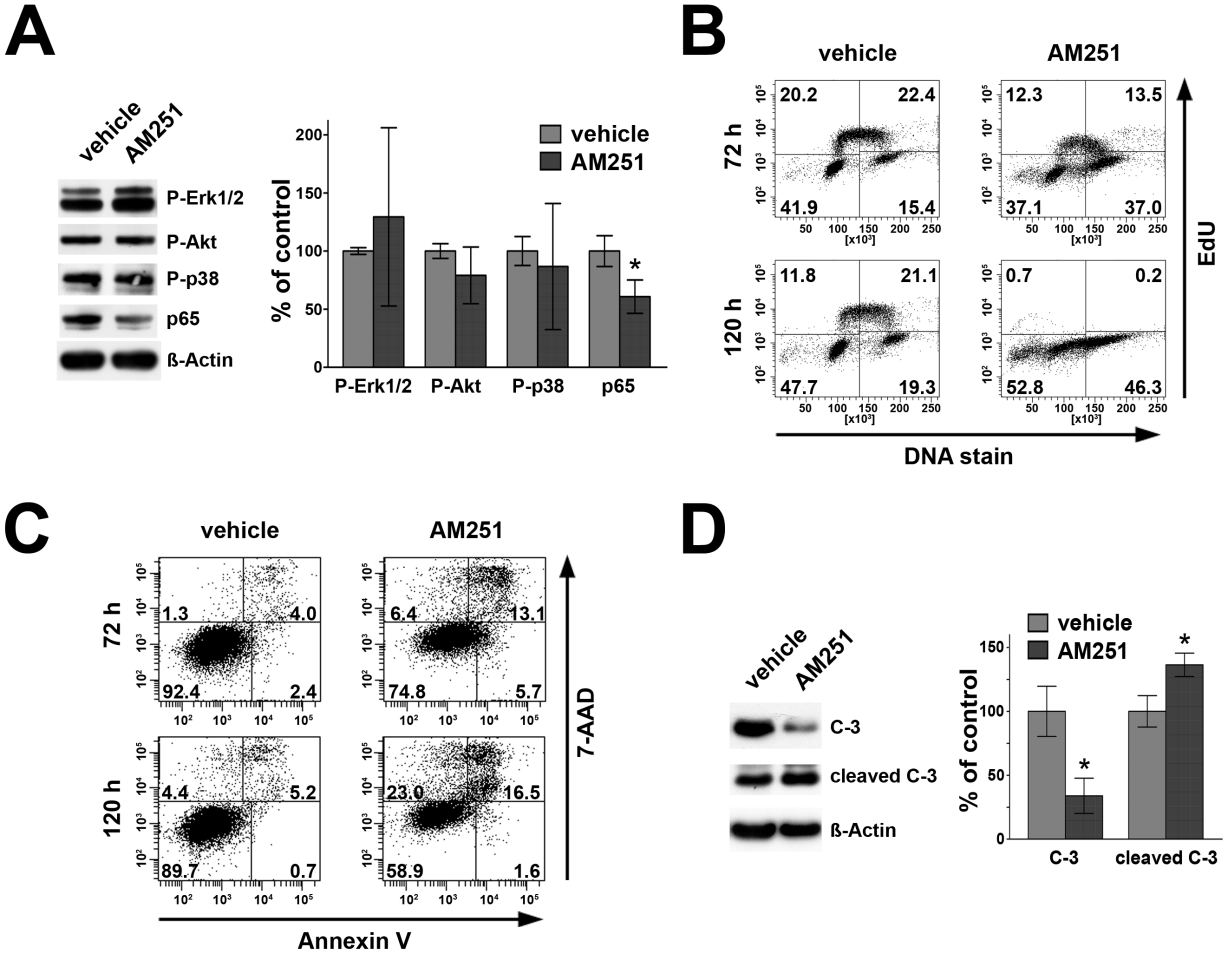
Effects of CB_1_ inhibition on signal transduction, p65-level, cell cycle profile and apoptotic populations in L428 cells. L428 cells were treated with 10 µM AM251. A) Western blot analysis of crude cell extracts showing a reduction of p65 whereas P-Erk1/2, P-Akt and P-p38 MAPK were not significantly altered compared to vehicle. B) Cell cycle analysis using EdU/DNA-stain and flow cytometric analysis showed strong decline of cells in S-phase and relative increase of cells in G2M phase. C) AnnexinV/7-AAD staining and subsequent flow-cytometric analysis revealed that after 72 h and 120 h of AM251-treatment, the number of vital cells decreased and apoptotic, necrotic and dead fractions were elevated. D) Processing of caspase-3 in a representative Western blot and its statistical analyses of L428 cells treated with AM251 for 96 h. AM251-treatment resulted in higher amounts of cleaved of caspase-3 (cleaved C-3) accompanied by a decrease of full length caspase-3 (C-3). Values represent means ± SD of 3 independent experiments.

To further analyze the nature of AM251 mediated reduction of viability in cHL cell lines, relative protein levels of P-Erk1/2, P-Akt, P-p38 MAPK and p65 were determined in L428 cells. Moreover, cell cycle analyses were performed and apoptotic cell demise was quantitatively detected (Figure 4). Treatment with 10 µM AM251 for 24 h did not affect phosphorylation of Erk1/2 (129.4±76.7%, p>0.05), Akt (79.1±24.4%, p>0.05) and p38 MAPK (86.7±54.2%, p>0.05) when compared to vehicles. However, significant reduction of p65 levels in crude cell extracts was seen after treatment with AM251 (60.8±14.3%, p<0.0001) (Figure 4A).

To resolve the relative changes of G1, early-S, late-S and G2M phases of cell cycle after AM251 treatment (10 µM) of L428 cells, EdU and DNA-specific staining was performed followed by flow cytometric analysis. After 72h and 120h L428 cells were stained with EdU and pacific blue (DNA stain). After 72 h, the number of cells in early-S changed from 20.2% (vehicle) to 12.3% (AM251), in late-S from 22.4% to 13.5%, in G1 from 41.9% to 37.1% and in G2M from 15.4% to 37.0%, respectively. After 120 h the population of cells changed in early-S from 11.8% (vehicle) to 0.7% (AM251), in late-S from 21.1% to 0.2%, in G1 from 47.7% to 52.8% and in G2M from 19.3% to 46.3%, respectively (Figure 4B). Compared to the effects of AM251, the distribution of cells in all four phases was only slightly changed after treatment with CB_1_ selective agonist ACEA for 72 h and 120 h (Figure S4B).

To analyze changes in apoptotic, necrotic and dead cell populations after AM251 treatment, flow cytometric analyses of L428 cells were carried out after 72 h and 120 h. After application of 10 µM AM251, L428 cells were stained with AnnexinV and 7-AAD. After 72 h, the number of vital cells (AnnV^−^/7AAD^−^) decreased from 92.4% (vehicle) to 74.8% in AM251 treated samples. The apoptotic (AnnV^+^/7AAD^−^) population was elevated under AM251-treatment (5.7%) compared to control conditions (2.4%). The necrotic (AnnV^+^/7AAD^+^) and dead fractions (AnnV^−^/7AAD^+^) were elevated (13.1 and 6.4%, respectively) compared to vehicle treated samples (4.0 and 1.3%, respectively). After 120 h, the viable population of controls (89.7%) was reduced after application of AM251 (58.9%). The apoptotic population was increased from 0.7 to 1.6%, necrotic cells from 5.2 to 16.5% and dead cells from 4.4 to 23% each with AM251 compared to controls, respectively (Figure 4C). The proportions of all four populations (vital, apoptotic, necrotic and dead) were not shifted when CB_1_ selective agonist ACEA was applied for 72 h and 120 h (Figure S4C).

To prove whether down stream members of apoptosis pathways were activated, the effects of AM251 treatment on caspase-3 cleavage were evaluated. In comparison to vehicle controls, application of AM251 (10 µM) for 96 h resulted in a decline of full length caspase-3 (33.9±13.8%, p<0.01) and in parallel to an induction of cleaved caspase-3 (136.4±9.2%, p<0.05, Figure 4D).

## Discussion

Involvement of CB_1_ in cell survival has been described in several types of cancer models but the functional relevance of CB_1_ in Hodgkin lymphoma has not been studied to date. In the present study, we report the abundance and anti-apoptotic role of CB_1_ in classical Hodgkin lymphoma.

The presence of functional CB_1_-protein has been reported in prostate cancer and hepatocellular carcinoma [18], [36]. In prostate cancer cells, higher amounts of CB_1_ were found when compared to their benign counterparts and high CB_1_-immunoreactivity correlated with severity of the disease [17]. Contrarily, elevated cannabinoid receptor expression in hepatocellular carcinoma was associated with improved prognosis [18]. Prognostic relevance of CB_1_ expression levels in lymphoid neoplasms such as HL remains to be determined.

CB_1_ protein is located at the plasma membrane, as well as in intracellular vesicles such as lysosomes. Intracellular CB_1_ receptors are associated with heterotrimeric G proteins, functional and able to mediate signal transduction [37]. As shown in other malignancies such as prostate carcinoma, we also found an intracellular and perinuclear CB_1_-staining pattern in tumor cells of HL. The subcellular localization of CB_1_ might further hint to internalization upon binding highly lipophilic endogenous ligands such as arachidonoylethanolamide (AEA) or 2-arachidonoylglycerol (2-AG) produced by HRS cells themselves or the surrounding reactive infiltrate.

Since blocking of CB_1_ resulted in a decline in viability of L428 cells, one might hypothesize that this GPCR is a survival factor for HRS cells and, in conclusion, its activation promotes tumor cell growth. In fact, involvement of activated CB_1_ signaling in liver regeneration was recently demonstrated in mice after partial hepatectomy via upregulation of cell cycle regulators [38]. In HL cells, however, application of CB_1_ agonist ACEA showed only marginal effects on apoptotic parameters in HL cells and even led to a slight decrease of cell viability. CB_1_ might act as a promoter of cell growth in HL cells. Endogenous agonists (endocannabinoids) such as AEA or 2-AG in the culture media might bind and activate CB_1_ in these cells, leaving the addition of synthetic agonists with adverse effects. Such a possible role for paracrine or even autocrine action of endogenous cannabinoids in HL is yet to be determined.

In colorectal cancer, the effects of CB_1_-specific treatment on cell viability are controversial. It was shown that CB_1_-activation of colon carcinoma cells resulted in inhibition of growth [39]. Santoro and colleagues reported on increased cell death of colon cancer cells using the CB_1_-antagonist SR141716 [19]. In rhabdomyosarcoma, increased CB_1_ expression was associated with enhanced proliferation and invasion which was blocked by application of CB_1_ antagonist/inverse agonist AM251 [40].

In breast cancer cell lines treated with the plant derived cannabinoid and CB_1_-agonist delta-9-tetrahydrocannabinol (THC), no alteration of cell viability was detected *in vitro*, but when the same cells were transplanted into mice, enhanced tumor growth was observed. In that case, the THC-mediated suppression of Th1-specific immune response in the animals was proposed to be responsible for enhanced growth *in vivo* [41]. The functional relevance of the endocannabinoid system in reactive immune cells surrounding HRS-cells needs further investigation in HL, with special regard to the fact that these specific reactive immune cells surrounding HRS-cells represent CB_1_ negative immune cells *in vivo*. Putative endocannabinoids might act via cannabinoid receptor 2 (CB_2_) since CB_2_ is known to be predominantly located in immune cells modulating immune cell migration and cytokine release [42].

Since we found a predominant expression of CB_1_ protein in lysates of HL derived cell lines, we subsequently analyzed the effects of pharmacological activation and inhibition of CB_1_ in HL derived cells expressing a relatively high amount of CB_1_. ACEA did not alter cell cycle or apoptotic parameter in flow-cytometric analyses. However, a striking decrease of cell viability down to 11% (L428), 44% (L540) and 13% (KM-H2) compared to control levels was observed upon application of 10 µM of CB_1_ inverse agonist AM251. A Striking reduction of L428 cells in S-phase was seen after inhibition of CB_1_. Although the effects of highest doses of CB_1_-agonist ACEA were significant on cell viability, they were not compelling, since 83% of L428 cells were still viable.

Expression of *Cnr1* in B- and T-cell NHL has earlier been described [43]-[45]. In line with these data, we also demonstrate *Cnr1* in B-NHL cell line Karpas 422 at mRNA as well as CB_1_ protein level. Unlike in HL cells, viability was not impaired after pharmacological inhibition of CB_1_. We conclude that the effects of AM251 on viability used in HL cells are not of unspecific toxic nature and hypothesize that the B-NHL cell line Karpas 422, compared to HL tumor cells, might use other intrinsic mechanisms bypassing CB_1_ dependent cell death.

Recently, another target of several CB_1_-antagonists was uncovered as AM251 was demonstrated to bind and activate the orphan receptor GPR55 [35]. To exclude GPR55 as a mediator of the effects observed after AM251 treatment, we performed viability assays using LPI, a ligand highly specific to GPR55 [46]. A significant decrease of L428 cell viability of 6% was detected with LPI which was marginal when compared to the effects of AM251 (89% reduction). Hence, the observed effects of AM251 on viability of L428 cells were most probably due to inhibition of CB_1_ rather than activation of GPR55.

Previously, we reported on aberrant expression and activation of certain receptor tyrosine kinases (RTK) in cases of HL. The constitutive activation of downstream signaling cascades was found to be an important survival-factor for HRS-cells [47], [48]. To determine whether RTK-signaling is involved in the observed reduction of cell viability after CB_1_-inhibition, we analyzed the effects of AM251 on phosphorylation of Akt and Erk1/2, two downstream targets within RTK-signaling pathways [49]. However, no significant change in phosphorylation of Akt at Ser473 or Erk1/2 at Thr202/Tyr204 was observed.

A crucial survival factor of HRS-cells in HL cases is the transcription factor p65 [50]. Aberrant activation of this transcription factor is a central mechanism to bypass apoptosis [32]. Activation of CB_1_ by THC was shown to increase the activity of p65 [51]. After CB_1_ antagonization, we found a remarkable decrease of p65-levels in L428 cells. Since others showed an induction of apoptosis of HRS-cells after knock-down of p65 [31], increased cell demise after inhibition of CB_1_ may be due to decreased p65-levels.

In conclusion, our data reveal that CB_1_ expression is a common feature of HRS-cells in cHL and suggest its antagonization as a possible novel strategy for specific pharmacological treatment of HL.

## Supporting Information

Figure S1 **Preabsorption of CB1-specific antibody.** Staining of human hippocampus slices and a case of NS with CB_1_-antibody and CB_1_-antibody incubated for 3 hours with CB_1_-immunizing peptide. In the Cornu ammonis region and in the hilar zone, some neurons displayed perinuclear positivity. Further, the neuropil of the hilar zone showed strong granulated CB_1_ abundance. The cytosol of HRS cells was stained positive for CB_1_. The CB_1_ positive structures in the hippocampus and the cHL case lost their immunoreactivity after preincubation with the corresponding peptide. Bars =  20 µm.
(TIF)

Figure S2 **Western blot analyses.** N-terminal CB_1_ Western blot of HL cell lines KMH2 and L428 with preabsorption using CB_1_ immunizing peptide.
(TIF)

Figure S3 **CB_1_-immunoreactivity in NLPHL and B-NHL subentities.** Analysis of 153 B-cell lymphoma cases stained with a N-terminal CB1-antibody. In total, cHL cases were positive in 83.7%. The cHL sub-entities NS and MC were positive in 90.1% and 75%, respectively. None of the NLPHL cases were found positive. In B-NHL subentities, 0% of MCL, 5.3% of MZL, 11.5% of DLBCL, 0% of FL and 0% of B-CLL cases were positive for CB1. Cases of NLPHL, DLBCL, FL, MCL, MZL and B-CLL were stained against CB_1_ (brown). Note that tumor cells of each entity (arrows) are mostly negative for CB_1_ whereas only a few non-neoplastic reactive cells (arrow heads) show a positive immunoreaction for CB_1_. Bars =  20 µm
(TIF)

Figure S4 **Effects of CB1 agonist ACEA and GPR55 agonist LPI on lymphoma derived cell lines.** A) Cell viability was determined in L428 and Karpas 422 cells treated with the indicated concentrations of ACEA for 120 h using the MTT-assay. When compared to vehicles, ACEA did not reduce the number of vital cells at 3 µM significantly (p>0.05) but at 10 µM (p<0.05). Administration of maximal dose of ACEA did not change the viability of Karpas 422 (p>0.05). The GPR55 specific agonist LPI slightly reduced viability of L428 cells at 10 µM (p<0.01). Values represent means ± SD. B) ACEA treated L428 cells and cell cycle proportions after 72 h and 120 h as revealed from EdU/nuclear stain and subsequent flow cytometric analysis. C) L428 cells were stained with AnnexinV/7-AAD. Subsequent flow-cytometric analysis revealed slight changes after 72 h of treatment with 10 µM ACEA.
(TIF)
