# Supplementary figures and images for: Expression and Functional Relevance of Cannabinoid Receptor 1 in Hodgkin Lymphoma
Source: PLoS One. 2013 Dec 9;8(12):e81675. doi: 10.1371/journal.pone.0081675 (PMC3857220; doi:10.1371/journal.pone.0081675)

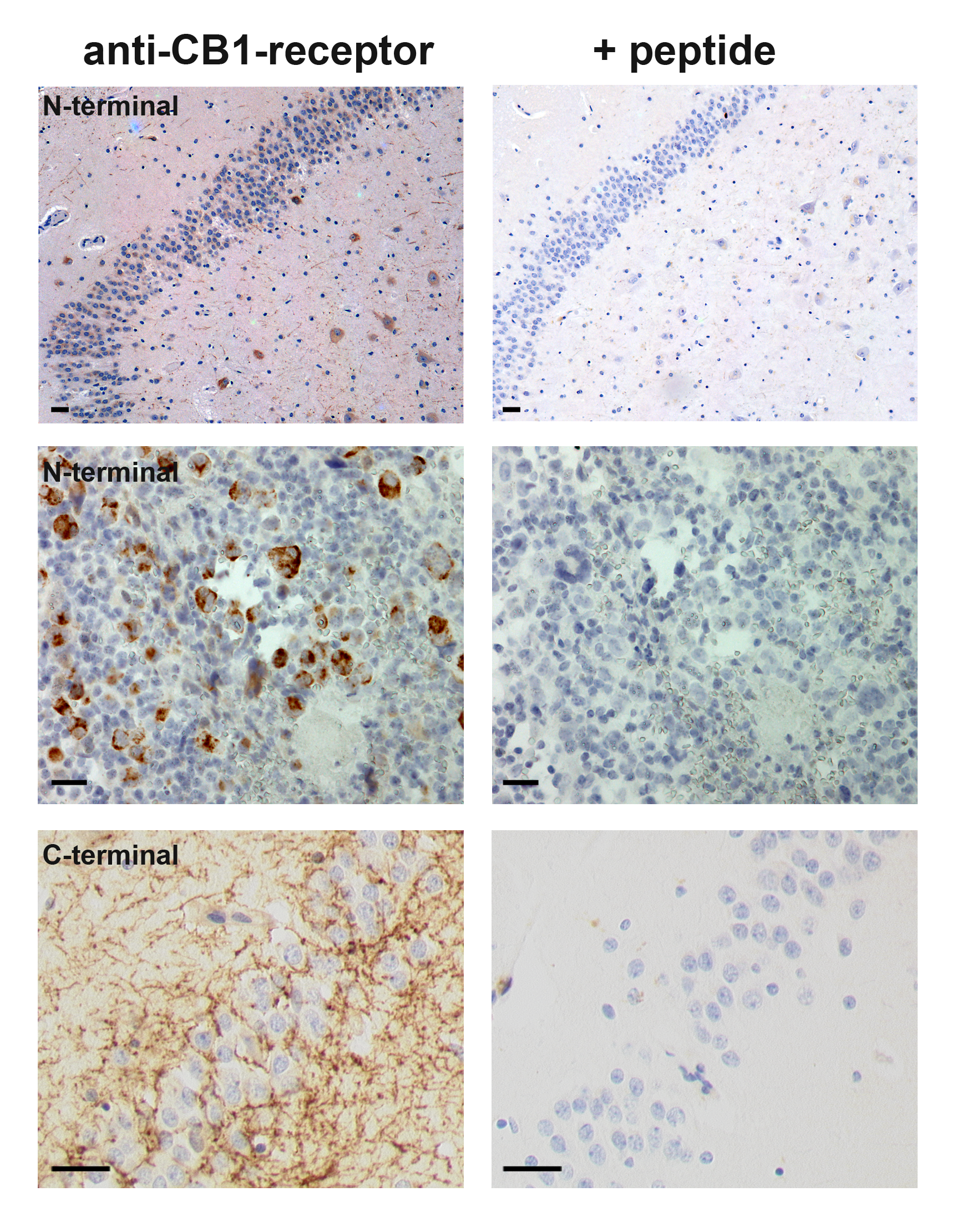

Supplement: Figure S1 — Preabsorption of CB1-specific antibody. Staining of human hippocampus slices and a case of NS with CB1-antibody and CB1-antibody incubated for 3 hours with CB1-immunizing peptide. In the Cornu ammonis region and in the hilar zone, some neurons displayed perinuclear positivity. Further, the neuropil of the hilar zone showed strong granulated CB1 abundance. The cytosol of HRS cells was stained positive for CB1. The CB1 positive structures in the hippocampus and the cHL case lost their immunoreactivity after preincubation with the corresponding peptide. Bars = 20 µm. (TIF) [file pone.0081675.s001.tif]

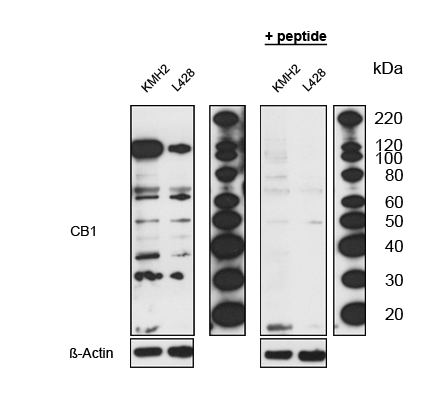

Supplement: Figure S2 — Western blot analyses. N-terminal CB1 Western blot of HL cell lines KMH2 and L428 with preabsorption using CB1 immunizing peptide. (TIF) [file pone.0081675.s002.tif]

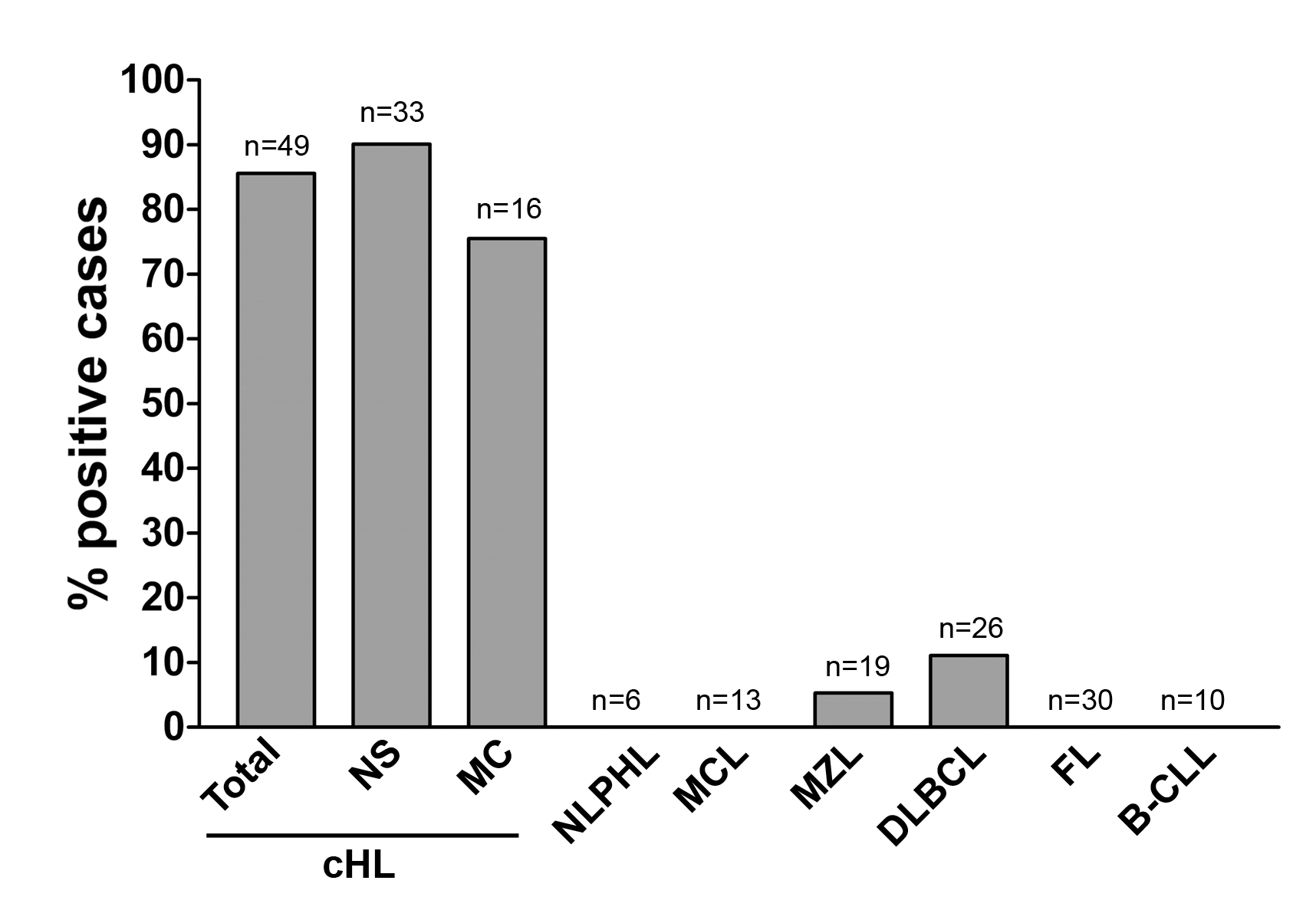

Supplement: Figure S3 — CB1-immunoreactivity in NLPHL and B-NHL subentities. Analysis of 153 B-cell lymphoma cases stained with a N-terminal CB1-antibody. In total, cHL cases were positive in 83.7%. The cHL sub-entities NS and MC were positive in 90.1% and 75%, respectively. None of the NLPHL cases were found positive. In B-NHL subentities, 0% of MCL, 5.3% of MZL, 11.5% of DLBCL, 0% of FL and 0% of B-CLL cases were positive for CB1. Cases of NLPHL, DLBCL, FL, MCL, MZL and B-CLL were stained against CB1 (brown). Note that tumor cells of each entity (arrows) are mostly negative for CB1 whereas only a few non-neoplastic reactive cells (arrow heads) show a positive immunoreaction for CB1. Bars = 20 µm (TIF) [file pone.0081675.s003.tif]

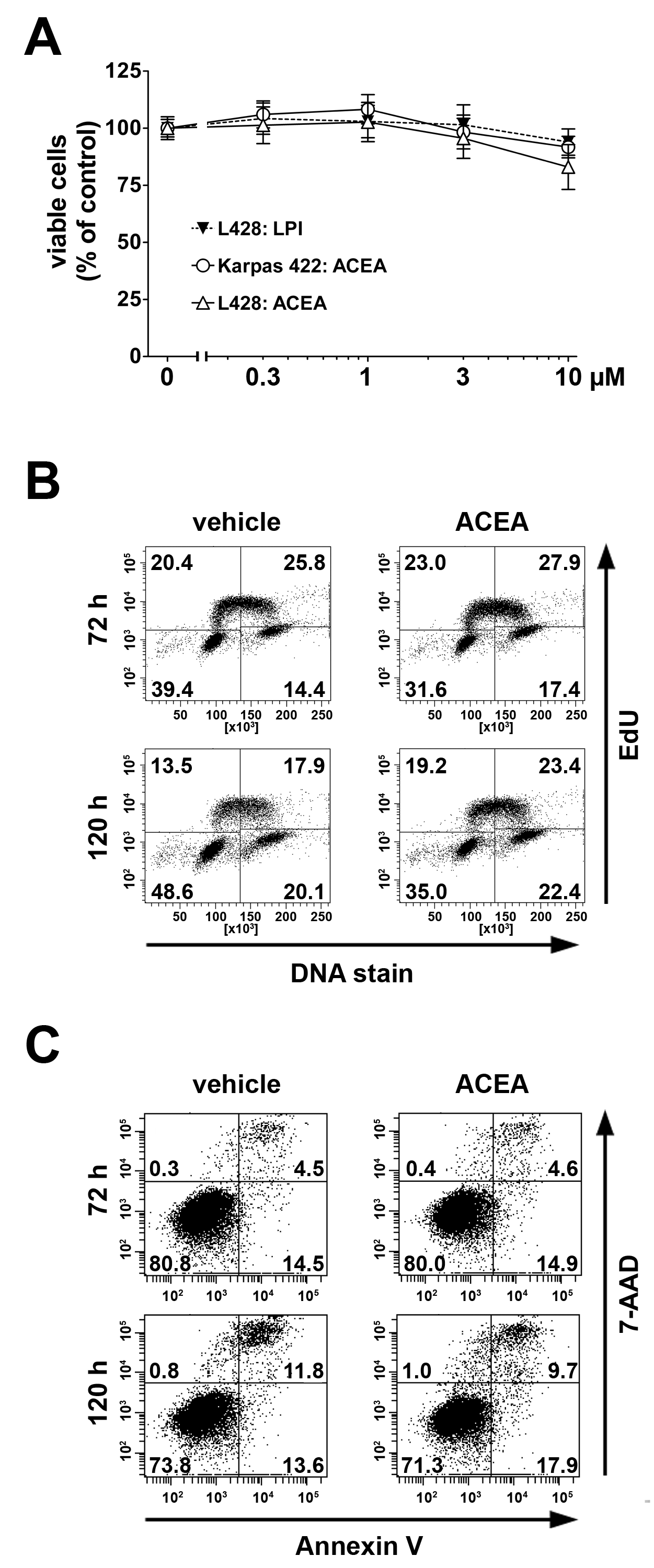

Supplement: Figure S4 — Effects of CB1 agonist ACEA and GPR55 agonist LPI on lymphoma derived cell lines. A) Cell viability was determined in L428 and Karpas 422 cells treated with the indicated concentrations of ACEA for 120 h using the MTT-assay. When compared to vehicles, ACEA did not reduce the number of vital cells at 3 µM significantly (p>0.05) but at 10 µM (p<0.05). Administration of maximal dose of ACEA did not change the viability of Karpas 422 (p>0.05). The GPR55 specific agonist LPI slightly reduced viability of L428 cells at 10 µM (p<0.01). Values represent means ± SD. B) ACEA treated L428 cells and cell cycle proportions after 72 h and 120 h as revealed from EdU/nuclear stain and subsequent flow cytometric analysis. C) L428 cells were stained with AnnexinV/7-AAD. Subsequent flow-cytometric analysis revealed slight changes after 72 h of treatment with 10 µM ACEA. (TIF) [file pone.0081675.s004.tif]
